# Efficacy of cognitive behavioral therapy on mood and quality of life for patients with COVID-19: A protocol for systematic review and meta-analysis

**DOI:** 10.1097/MD.0000000000025512

**Published:** 2021-04-16

**Authors:** Youxiang Zheng, Lu Wang, Yimei Zhu, Yan Zeng

**Affiliations:** a Department of Psychiatry; b Department of Infectious Disease, Xiantao First People's Hospital Affiliated to Yangtze University, Hubei 433000, China.

**Keywords:** COVID-19, cognitive behavioral therapy, review, meta-analysis, protocol

## Abstract

**Background:**

There is no study that has conducted a review or meta-analysis investigating a cognitive behavioral therapy (CBT) intervention to patients with COVID19, with the aim of improving their psychological health. Therefore, in order to provide new evidence-based medical evidence for clinical treatment, we undertook a systematic review and meta-analysis to assess the effectiveness of CBT in relieving patients’ psychological distress and improving quality of life during the COVID-19 epidemic.

**Methods:**

Seven electronic databases including Web of Science, Embase, PubMed, Wanfang Data, Scopus, Science Direct, Cochrane Library will be searched in April 2021 by 2 independent reviewers. For search on PubMed, the following search terms will be used: “COVID-19, 2019 Coronavirus Disease, 2019-nCoV, cognitive behavioral therapy, CBT, cognitive behavioral treatment.” In order to achieve a consistency (at least 80%) of extracted items, the data extractors will extract data from a sample of eligible studies. The outcomes include any rating scale describing stress, mood, and quality of life. Review Manager software (v 5.4; Cochrane Collaboration) will be used for the meta-analysis. Two independent reviewers will assess the risk of bias of the included studies at study level. Any disagreements will be discussed and resolved in discussion with a third reviewer.

**Results:**

The results of our review will be reported strictly following the PRISMA criteria.

**Conclusions:**

The review will add to the existing literature by showing compelling evidence and improved guidance in clinic settings.

**OSF registration number:**

10.17605/OSF.IO/DCRPJ.

**Ethics and dissemination:**

Ethical approval and patient consent are not required because this study is a literature-based study. This systematic review and meta-analysis will be published in a peer-reviewed journal.

## 1 Introduction

The 2019 Coronavirus Disease (COVID-19) pandemic has had serious health implications that extend wellbeyond symptoms of the virus. Mental health problems, including insomnia, post-traumatic stress, and depressive symptoms, have been observed in the context of COVID-19 and were documented in previous epidemics (e.g., SARS, MERS).^[1]^ Therefore, COVID-related disability and mortality will include damage from mental illness, which is already a major contributor to the global burden of disease. Public health interventions which aim at reducing the burden of COVID-related disease should include interventions that promote mental resilience.^[2–4]^

Cognitive behavioral therapy (CBT), as an evidence-based psychotherapy developed for health care workers, has been widely used for the treatment and prevention of mental and physical distress in the community and in hospital.^[5]^ CBT is a series of methods, including cognitive reconstruction, behavioral change and social support, designed to help individuals identify stress levels and change negative cognitive beliefs and behaviors, reduce or eliminate symptoms of psychological distress, and further help individuals return to normal life in terms of psychological and social functions.^[6–8]^

CBT has been found to be effective in preventing burnout among health care workers in stressful situations outside of the current COVID-19 pandemic. In addition, there is evidence that CBT can be effective in preventing many psychiatric disorders in high-risk populations, such as posttraumatic stress disorder and depression.^[9–11]^ However, to the best of our knowledge, there is no study that has conducted a review or meta-analysis investigating a CBT intervention to patients with COVID-19, with the aim of improving their psychological health. Therefore, in order to provide new evidence-based medical evidence for clinical treatment, we undertook a systematic review and meta-analysis to assess the effectiveness of CBT in relieving patients’ psychological distress and improving quality of life during the COVID-19 epidemic.

## 2 Materials and methods

### 2.1 Protocol registration

The prospective registration has been approved by the Open Science Framework (OSF) registries, and the registration number is 10.17605/OSF.IO/DCRPJ. The protocol was written following the Preferred Reporting Items for Systematic Reviews and Meta-Analyses Protocols (PRISMA-P) statement guidelines.

### 2.2 Searching strategy

**Figure 1:**
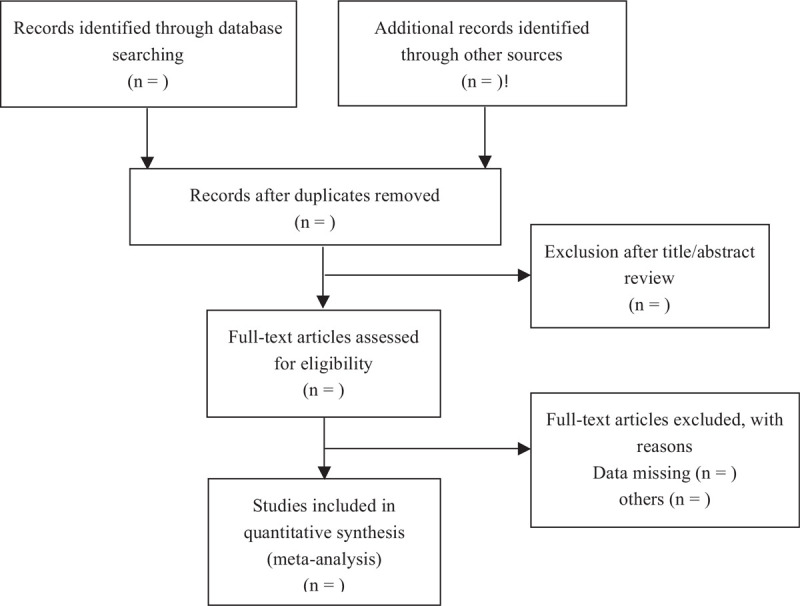
PRISMA Flow diagram describing the selection process for relevant clinical trials used in this meta-analysis.

Seven electronic databases including Web of Science, Embase, PubMed, Wanfang Data, Scopus, Science Direct, Cochrane Library will be searched in April 2021 by 2 independent reviewers. For search on PubMed, the following search terms will be used: “COVID-19, 2019 Coronavirus Disease, 2019-nCoV, cognitive behavioral therapy, CBT, cognitive behavioral treatment.” To minimize the risk of publication bias, we will conduct a comprehensive search that included strategies to find published and unpublished studies. The reference lists of the included studies will also be checked for additional studies that are not identified with the database search. There is no restriction in the dates of publication or language in the search. No ethical approval is required in our study because all analyses will be based on aggregate data from previously published studies (Fig. 1).

### 2.3 Eligibility criteria

Study included in this review has to meet all of the following inclusion criteria in the PICOS order:

(1) population: patients with COVID-19;
(2) intervention group (group 1): routine treatment with additional CBT;
(3) comparison group (group 2): routine treatment;
(4) outcome measures: any rating scale describing stress, mood, and quality of life;
(5) study design: randomized controlled trial or observational study.

Biomechanical studies, in vitro studies, review articles, techniques, case reports, letters to the editor, and editorials are excluded.

### 2.4 Study selection

The first author will conduct a preliminary screening based on the title to eliminate any research not related to the topic. A log of excluded studies is kept with the rationale for exclusion. Subsequently, all remaining abstracts will be reviewed by the primary author, and the selection criteria are applied. Studies identified for full text review will be evaluated by 2 authors for inclusion in the study. Disagreements will be resolved through a discussion with a third review author. Journal titles and authors’ names will be not glossed over in the research selection process. A manual search of the bibliographies of included studies is performed to ensure that the overall search was comprehensive and complete.

### 2.5 Data extraction

In order to achieve a consistency (at least 80%) of extracted items, the data extractors will extract data from a sample of eligible studies. Results of the pilot extraction will be discussed among review authors and extractors. Two independent reviewers will extract data with a predefined extraction template, which includes the following items: study characteristics such as the first author, publication year, study design, follow-up period; patient demographic details such as patients’ number, average age, and gender ratio. The outcomes include any rating scale describing stress, mood, and quality of life. The original authors will be contacted to request missing data where necessary. Extracted information will be cross-checked by 2 independent reviewers. Any disagreements will be discussed and resolved in discussion with a third reviewer.

### 2.6 Statistical analysis

Review Manager software (v 5.4; Cochrane Collaboration) will be used for the meta-analysis. Continuous variables are extracted and analyzed to mean value ± SD. Standardized mean differences with a 95% confidence interval are assessed for continuous outcomes. The heterogeneity is assessed by using the Q test and *I*^2^ statistic. An *I*^2^ value of <25% is chosen to represent low heterogeneity and an *I*^2^ value of >75% to indicate high heterogeneity. All outcomes are pooled on random-effect model. A *P* value of <.05 is considered to be statistically significant.

### 2.7 Quality evaluation

In order to achieve a consistency (at least 80%) of risk of bias assessment, the risk of bias assessors will pre-assess a sample of eligible studies. Results of the pilot risk of bias will be discussed among review authors and assessors. Two independent reviewers will assess the risk of bias of the included studies at study level. We will follow the guidance in the latest version of Cochrane Handbook for systematic reviews of interventions when choosing and using tools to assessing risk of bias for randomized trials (version 2 of the Cochrane risk of bias tool for randomized trials, RoB 2) and nonrandomized trials (the Risk Of Bias In Non-randomized Studies of Interventions, ROBINS-I tool). Any disagreements will be discussed and resolved in discussion with a third reviewer. Studies with a high risk of bias or unclear bias will be given less weight in our data synthesis.

## 3 Discussion

There is evidence that CBT can be effective in preventing many psychiatric disorders in high-risk populations, such as post-traumatic stress disorder and depression. However, to the best of our knowledge, there is no study that has conducted a review or meta-analysis investigating a CBT intervention to patients with COVID19, with the aim of improving their psychological health. Therefore, in order to provide new evidence-based medical evidence for clinical treatment, we undertook a systematic review and meta-analysis to assess the effectiveness of CBT in relieving patients’ psychological distress and improving quality of life during the COVID-19 epidemic. We believe that patients who had close communication with family and friends and receive encouragement from medical staff helps them improve their psychological health. Previous studies have indicated that these strategies can enhance patient's self-confidence and reduce the psychological stress response caused by epidemics such as SARS and COVID-19. It can further have a great impact in promoting the physical and psychological health of patients. For this study, our review process will be very rigorous. And this article is a protocol of the systematic review and meta-analysis, which presents the detailed description of review implement. The results of our review will be reported strictly following the PRISMA criteria and the review will add to the existing literature by showing compelling evidence and improved guidance in clinic settings.

## Author contributions

**Conceptualization:** Youxiang Zheng, Yimei Zhu, Lu Wang.

**Data curation:** Youxiang Zheng, Lu Wang.

**Formal analysis:** Youxiang Zheng, Lu Wang.

**Funding acquisition:** Yan Zeng.

**Investigation:** Youxiang Zheng, Lu Wang.

**Methodology:** Lu Wang, Yimei Zhu.

**Project administration:** Yan Zeng.

**Resources:** Yan Zeng.

**Software:** Yimei Zhu.

**Supervision:** Yan Zeng.

**Validation:** Lu Wang.

**Visualization:** Lu Wang.

**Writing – original draft:** Youxiang Zheng, Lu Wang.

**Writing – review & editing:** Yan Zeng.
